# Fabrication of Circuits on Flexible Substrates Using Conductive SU-8 for Sensing Applications

**DOI:** 10.3390/s17061420

**Published:** 2017-06-17

**Authors:** Carlos D. Gerardo, Edmond Cretu, Robert Rohling

**Affiliations:** 1 Department of Electrical and Computer Engineering, University of British Columbia, Vancouver, BC V6T 1Z4, Canada; edmondc@ece.ubc.ca (E.C.); rohling@ece.ubc.ca (R.R.); 2 Department of Mechanical Engineering, University of British Columbia, Vancouver, BC V6T 1Z4, Canada

**Keywords:** SU-8, silver nanoparticles, percolation threshold, planar inductors, passive filters, interconnections

## Abstract

This article describes a new low-cost rapid microfabrication technology for high-density interconnects and passive devices on flexible substrates for sensing applications. Silver nanoparticles with an average size of 80 nm were used to create a conductive SU-8 mixture with a concentration of wt 25%. The patterned structures after hard baking have a sheet resistance of 11.17 Ω/☐. This conductive SU-8 was used to pattern planar inductors, capacitors and interconnection lines on flexible Kapton film. The conductive SU-8 structures were used as a seed layer for a subsequent electroplating process to increase the conductivity of the devices. Examples of inductors, resistor-capacitor (RC) and inductor-capacitor (LC) circuits, interconnection lines and a near-field communication (NFC) antenna are presented as a demonstration. As an example of high-resolution miniaturization, we fabricated microinductors having line widths of 5 μm. Mechanical bending tests were successful down to a 5 mm radius. To the best of the authors’ knowledge, this is the first report of conductive SU-8 used to fabricate such planar devices and the first on flexible substrates. This is a proof of concept that this fabrication approach can be used as an alternative for microfabrication of planar passive devices on flexible substrates.

## 1. Introduction

Composite materials exhibit properties that cannot be found with metals, polymers or ceramics alone. In particular, metallic nanoparticles embedded in polymers have become increasingly popular because of their ability to maintain the electrical properties in the metallic range while maintaining the flexibility and some unique properties of polymers such as bio-compatibility, chemical resistivity, optical transparency, photo-sensitivity, etc. These composites materials do not have the drawbacks of pure metals such as high density, low chemical resistance and complex manufacturing process.

Photoresists are good candidates to be mixed with these metallic particles; they are chemically inert, mechanically stable and can be easily spin coated. Photoresists, such as epoxy-based SU-8 [1], have a broad range of use, such as fabricating high-aspect ratio structures, molds and microfluidic channels. The incorporation of particles in the nanoscale range can be used to tailor specific electrical, optical, or mechanical properties, broadening the applications for this material.

The electrical conductivity of these composite materials depends strongly on the type of filler and the proportion and average size of the particles. As outlined by [2], the larger surface area of small particles allows conductive SU-8 to be obtained with a lower concentration of particles. The point in which the polymer matrix with fillers starts to become electrically conductive is known as the percolation threshold [3]. After this point, an increased amount of conductive fillers in the polymer matrix leads to a smaller increase in the electrical conductivity.

Conductive SU-8 can be obtained by mixing silver nanoparticles [2,4], carbon black powder [5,6], single and multi-walled carbon nanotubes [7,8,9], graphene [10], conductive organic components [11], gold and even diamondoids [12]. Depending on the type of filler the mechanical, optical and electrical properties of the fabricated device are affected [12].

One of the main limitations of the addition of metal nano-particles in SU-8 is the increase of light absorbance, reducing the photo-polymerization depth. In this work, silver was selected as metallic filler for its low absorption in the near UV range, as outlined in [13], allowing us to obtain thin conductive traces. Moreover, silver nanoparticles have a low absorption rate at the irradiation wavelength (365 nm) in comparison to other metallic fillers or oxides.

Some of the reported applications of conductive SU-8 include strain sensors [14,15], pressure sensors [9], biochips for electrografting [6], and as conductive material for inkjet printable applications [8]. Preliminary work involving circuit interconnection lines has only been reported in [16], where the conductive mixture of silver and SU-8 was deposited using a scraper and then back-side exposed through a quartz substrate; the patterned traces had a resistance variation from 800 Ω to 10 kΩ due to the inhomogeneity of the composite structures. The fabrication of passive elements using functionalized (or conductive) SU-8 has not yet been reported in the literature.

In this article, we present a new fabrication approach to create planar passive devices and circuit interconnects with the possibility of miniaturization, and how this technique can lead to obtain highly conductive lines as small as 5 μm. This leads to the potential fabrication of sensing and communication devices such as [17]. We also explored the feasibility of fabricating planar resistors and capacitors on flexible substrates using our functionalized SU-8. This approach simplifies the standard commercial fabrication process of such devices, where they are normally fabricated by selectively etching metallic foils glued to flexible substrates. This technique also enables the fabrication of such devices on substrates that are sensitive to the conditions present in evaporation or sputtering systems for metal deposition; moreover, fewer fabrication steps and required equipment are necessary when compared to traditional lift-off metal patterning techniques, translating into lower fabrication costs.

Typically, the number of passive components in a printed circuit board (PCB) is higher than the number of active components such as operational amplifiers and integrated circuits; therefore, a high number of electrical interconnections are needed to wire these devices together, sometimes needing multiple layers for a proper connection. Embedded passive components can be fabricated between these layers to simplify the design and reduce the footprint. Further advantages include higher reliability, better electrical performance, decreased number of electrical interconnections and low cost [18]. Perhaps the most important advantage of having passive elements on flexible substrates is that they meet the needs of the emerging flexible wearable devices manufacturing industry, an area forecasted to grow almost 67% in the next four years [19].

Because of their unique mechanical properties after curing, SU-8 has been widely used as structural material for the fabrication of MEMS devices [20], allowing the creation of cantilevers, beam arrays and membranes. The final goal of our research is to create operational MEMS devices and circuit interconnections using conductive SU8, allowing a hybrid integration with commercial integrated circuits and components; therefore, we selected SU-8 as the material for passive components, circuit interconnections and the MEMS devices themselves. The results presented in this article represent a portion of the overall scope of this project. Some preliminary results have been published in [21], where suspended beams and membranes fabricated with conductive SU-8 are electrostatically actuated.

Being able to spin deposit and photo-pattern at high resolution conductive areas is of special interest for the microfabrication community, as it increases the flexibility in the fabrication process in areas of microelectromechanical systems (MEMS) and microfluidics, where temperature-sensitive materials and processes are used and they would be otherwise incompatible with conventional metal deposition processing. Moreover, adhesion of metals to polymers is poor, requiring adhesion promoters to be used prior to metal deposition [22].

Modern electronics often require surface mounted elements to be attached to flexible circuits. The current flexible electronics industry allows circuits to be built using interconnection lines as small as 25 μm using copper traces with a spacing of 50 μm between lines [23]. These parameters usually come from the restrictions involved in their fabrication process (deposition and patterning of masking layer, wet etching using aggressive chemicals, etc). Our approach for the fabrication of RC circuits involves three major steps: spin coating of the sample using functionalized SU-8, UV exposure and sample development. An extra electroplating stage is beneficial for structures requiring a very low resistance value, such as inductors, interconnection lines or radio frequency (RF) fractal antennas [24].

## 2. Materials and Methods

### 2.1. Functionalized SU-8 Preparation

Guided by the results obtained in [2,4], a formulation containing 25 wt% silver dispersed in SU-8 2005 was prepared by our research group. Silver nonparticles with an average size of 80 nm were purchased from ACS Materials (Medford, MA, USA) and SU-8 2005 was acquired from Microchem (Newton, MA, USA). An accurate concentration of the mixture was obtained using a sensitive microbalance to incorporate the desired weight percentage of silver nanoparticles into the SU-8.

The silver nanoparticles were gradually introduced into the SU-8 while a high-speed mixer was used to evenly disperse the particles and minimize silver aggregation. This agitation process created unwanted air bubbles, which were later removed by immersing the sample in an ultrasound bath for 30 min. To avoid any silver to sediment at the bottom of the vial, a spin bar and a magnetic stirrer at moderate speed was used to maintain a good dispersion of the particles in the sample prior spin coating. Some research groups use pre-treated silver nanoparticles to enhance dispersion in the mixture [3]; a few others add a small percentage of surfactant or extra solvents into the mixture to have a uniform distribution of nanoparticles [12].

The addition of silver nanoparticles into the SU-8 increases the viscosity of the mixture, increasing the expected film thickness. For example, the film thickness of ordinary SU-8 2005 spun at 1000 RPM on a silicon wafer was 7.2 μm, and it increased to 10.6 μm after the addition of silver particles for the same spinning conditions.

Several experiments were performed by varying the concentration of silver in the SU-8 with increments of 5%, where the percolation threshold was detected to be around 20%. After experimentation, we noticed some variation in the conductivity of the patterned traces in different locations of the same sample. We mitigated this phenomenon by using a slightly higher concentration of silver nanoparticles of 25% in the mixture. As a consequence, the conductivity became much more controllable, especially for fine traces; nevertheless, the minimum patterned features increased in size as well as the required exposure dose. Initial experiments revealed a standard variation in the sheet resistance of ±8.2 Ω/☐ when a concentration of 20% was used. After the concentration was increased to 25%, the standard variation in the sheet resistance dropped to ±5.8 Ω/☐. When the concentration of silver was 60%, it was not possible to achieve any patterned traces on the substrate even when high UV doses were used; at this point, the concentration of nanoparticles was so high that that the UV light could not penetrate the entire thickness of the layer so it could be adhered to the substrate.

### 2.2. Sample Processing

For the fabrication of planar inductors and capacitors, a piece of Kapton film (DuPont, DE, USA) with a thickness of 137 μm was fixed to a silicon wafer for processing. Kapton was used because of its well known thermal resistance and electrical insulating properties; this particular thickness of Kapton was chosen as it allowed an easy manipulation of the sample without inducing any damage. The sample was later spin coated with our prepared functionalized SU-8 2005 at 1000 RPM for 30 s. A soft baking step on a hotplate at 95 °C for 5 min was sufficient to evaporate the cyclopentanone solvent present in the SU-8, leaving the sample ready to be exposed.

The photomask design was exposed using a maskless lithographic system SF-100 Xpress from Intelligent Micropatterning (St. Petersburg, FL, USA) with 0.6 μm resolution. The maskless lithographic system used for this project takes a photomask design in DXF (Drawing Exchange Format) and divides it into exposure frames of 1300 × 800 μm. During the exposure process, the frames are stitched by overlapping areas of 20 pixels per side. In order to avoid stitching defects, we adjusted the stitched area exposure with a grayscale value of 178 for the side areas and 155 for the corner areas according to [25], enhancing the surface uniformity.

After exposure, the sample was post-exposure baked for five additional minutes and then developed for 1 min in an SU-8 developer in a gentle agitation environment. A short rinse in acetone and isopropanol helped to remove silver residues from the substrate. In preliminary work, we noticed that, by spinning and baking a layer of ordinary SU-8 2005 before depositing the conductive SU-8 improved the adhesion of the fabricated devices, it also allowed us to achieve a smaller gap between features since the silver residues were able to be washed away more easily.

## 3. Experimental Results

**Figure 1:**
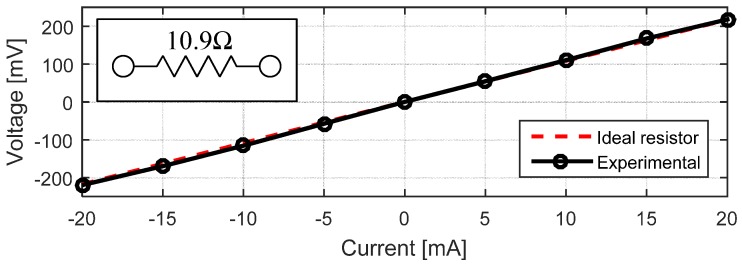
I–V graph showing a linear behavior for structures patterned using functionalized SU-8.

In order to calculate the sheet resistance of our conductive SU-8 films, we used a cloverleaf design following [26]. A four-probe measurement was used to measure the voltage drop on one side of the test structure due to a variation in the DC current flowing on the opposite side as shown in Figure 1. We determined that the sheet resistance of our conductive SU-8 layer (without electroplating) is 11.17 Ω/☐ which matches the results obtained by [3].

**Figure 2:**
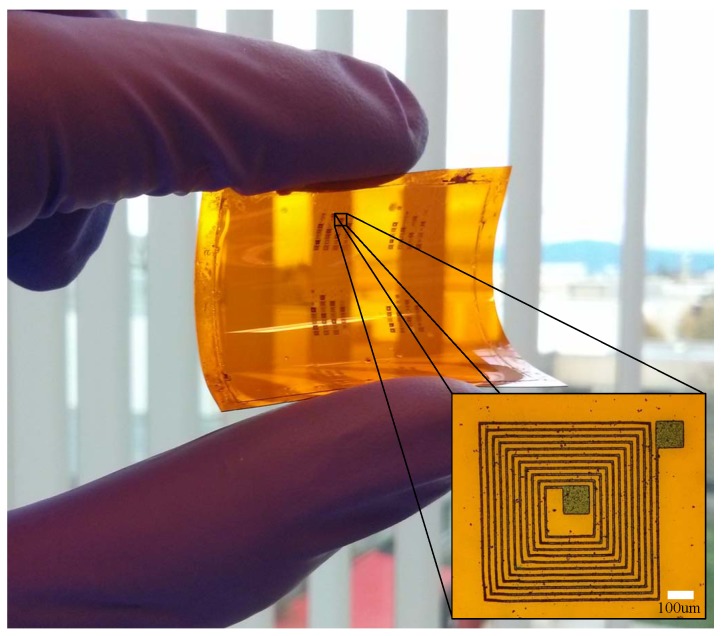
Array of microinductors fabricated using conductive SU-8 with the proposed fabrication technique. Microinductor with 5 μm lines and 20 μm spacing shown in the inset.

We fabricated planar resistors and capacitors using our functionalized SU-8 on Kapton films. The last step in our fabrication process is to hard bake the patterned features at 200 °C for 2 h to increase the conductivity of the traces as described in [3]. We successfully fabricated microinductors by electroplating copper on the SU-8 traces to increase the electrical conductivity. By using a maskless lithographic system to directly pattern the interconnection lines, instead of a masking layer for etching, we obtained conductive lines as small as 5 μm in width shown in Figure 2.

### 3.1. RC Filter Fabrication

Passive filters fabricated as planar devices are becoming increasingly popular for flexible circuits as an alternative of soldering surface-mounted devices.

**Figure 3:**
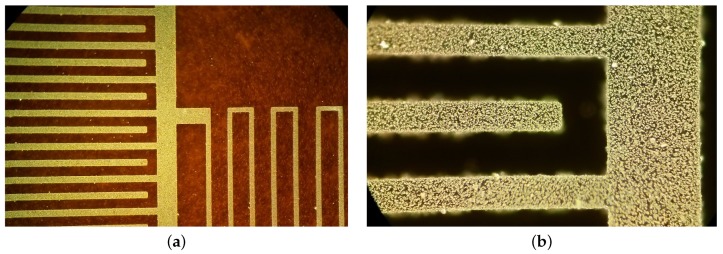
(**a**) RC filter showing the capacitor on the left and the resistance path on the right; (**b**) dark-field view of a capacitor finger showing the silver nanoparticles embedded in the SU-8 matrix.

We designed an RC array to be used as a low-pass filter built on a piece of Kapton film. The final resistor and capacitor are shown in Figure 3a. The silver nanoparticles dispersed in the SU-8 matrix can be seen in detail in Figure 3b.

**Figure 4:**
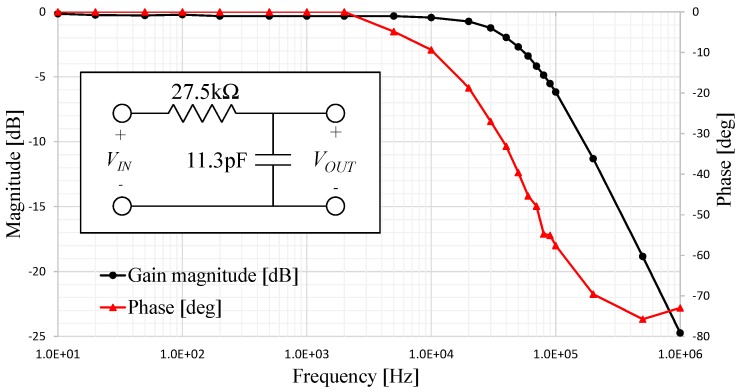
Experimental frequency response of a low-pass RC filter fabricated using conductive SU-8 on Kapton film.

We connected the RC circuit to act as a low-pass filter, a sinusoidal voltage with an amplitude of 10 V was used as input. The frequency response of the system is shown in Figure 4; where the measured cut-off frequency of this filter was 52 kHz, a 20 dB/dec attenuation clearly shows the behavior of a first-order system. The estimated values for resistance and capacitance including the interconnection cable parasitics are 270 kΩ and 11.33 pF, respectively.

**Figure 5:**
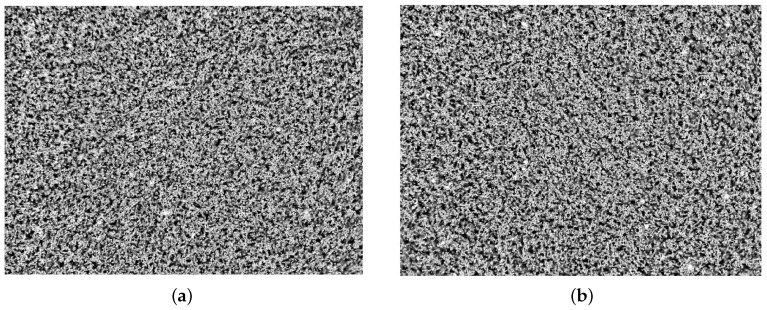
Dark-field microscope images showing the dispersion of silver nanoparticles in different locations within a sample (**a**) center of sample; (**b**) area close to the edge of sample.

To assess the silver particle distribution, we obtained dark-field images of unexposed conductive SU-8 in different regions of a sample, including the center and areas close to the edges. The silver particle density in the image was estimated using image processing techniques from [27], where the variation in density between different images was less than 3.5%. Figure 5 depicts the distribution of silver in the center of a sample compared to an area close to the edge.

### 3.2. Copper Electroplating

**Figure 6:**
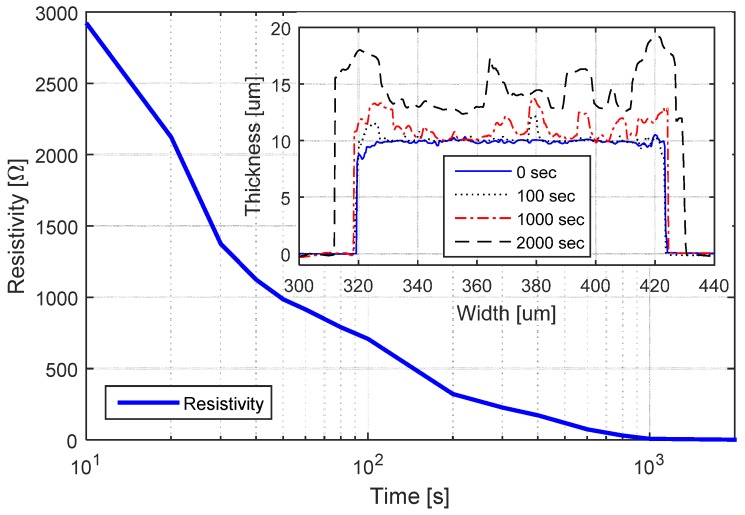
Resistivity variation of a 100 μm conductive SU-8 line for a 1 mA electroplating current. The cross-sectional view of the line is shown in the inset.

Conductive traces patterned using functionalized SU-8 can be used as a seed layer during an electroplating process to increase the electrical conductivity of the devices. To monitor the copper growth, we patterned a line with functionalized SU-8 measuring 100 μm in width by 10 mm in length; we then immersed our sample in an electroplating bath maintaining a current of 1mA. The electrical resistivity and the topography were measured over time. Figure 6 shows the rapid decreasing of the resistivity during the first minutes of electroplating; a cross-sectional view of the electroplated line is shown in the inset.

### 3.3. Inductor Fabrication

As it was mentioned before, a copper electroplating process allowed us to decrease the electrical resistivity of planar inductors fabricated with functionalized SU-8. A low series resistance is necessary to create inductors with high quality factors. The electroplating time and current intensity allows the tuning to the desired conductance.

**Figure 7:**
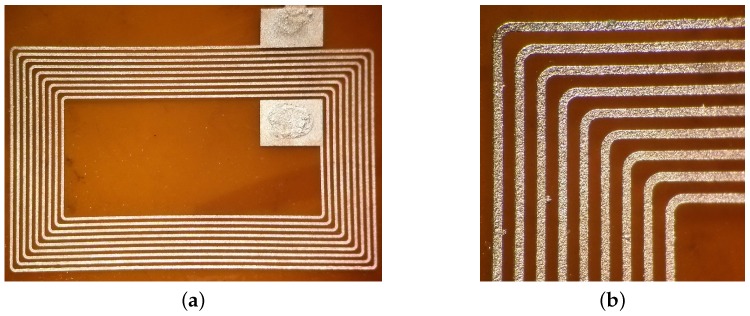
(**a**) NFC antenna fabricated with functionalized SU-8 as a seed layer after copper electroplating; (**b**) detailed view of electroplated traces.

As an example of this fabrication approach, we replicated the design of a commercially available NFC microantenna [28] using our functionalized SU-8; this particular design has 9 turns of 80 μm paths with 120 μm spacing. The sample was electroplated for 2.5 h maintaining a low current of 1 mA to improve the quality of the final copper layer. Figure 7 shows the final device as well as the details of the traces. Before electroplating, the sample had a series resistance of 34.6 kΩ; after electroplating, the electrical resistance dropped to 10.2 Ω.

**Figure 8:**
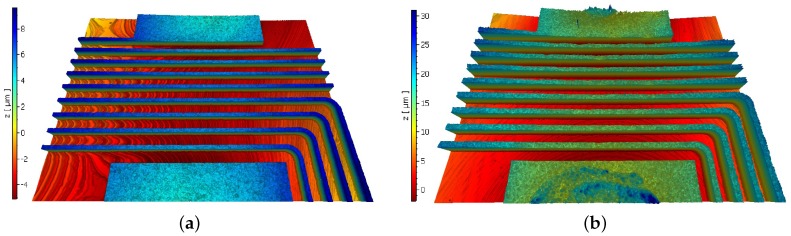
(**a**) Topography of replicated NFC antenna design before electroplating; (**b**) topography after electroplating.

The topography of the inductor traces was measured using a white light interferometer (Polytec, Irvine, CA, USA). Figure 8 shows images of the same traces before and after the electroplating process. The outermost terminal of the coil was connected to the cathode in the electroplating setup. The deposition of copper began closest to the negative terminal and gradually propagated towards the center of the coil, meaning that the outermost turns of the inductor are slightly thicker and wider than the inner ones: 3 μm is the maximum size difference in width.

**Figure 9:**
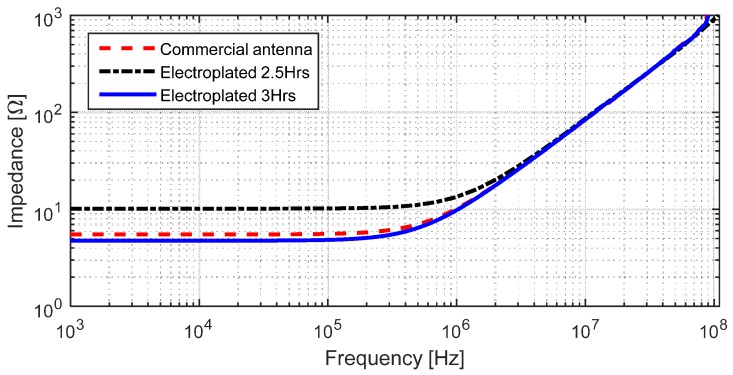
Frequency response of fabricated NFC antenna with electroplated copper on top of conductive SU-8.

The electrical impedance of the antenna was measured using an impedance analyzer Agilent E4294A (Santa Rosa, CA, USA). Figure 9 shows the comparison of the frequency response of the fabricated structure and the commercial NFC antenna. The measured inductance of the commercial version was 1.29 μH, while our inductor had a very similar value of 1.27 μH when electroplated for 2.5 h. To decrease the series resistance even further, the sample was electroplated one more time for a total of three hours, obtaining an inductance of 1.32 μH. The small discrepancy in the inductance values is likely due to the variation in geometry after the electroplating step; the tracks increased their width by 7.5 μm while the spacing between lines decreased. Moreover, the final thickness of our fabricated antenna is 18.5 μm, whereas the thickness of the commercial version is 70 μm. By controlling the electroplating time and current, we can tune the desired electrical conductivity of lines, adding flexibility to the fabrication process.

**Figure 10:**
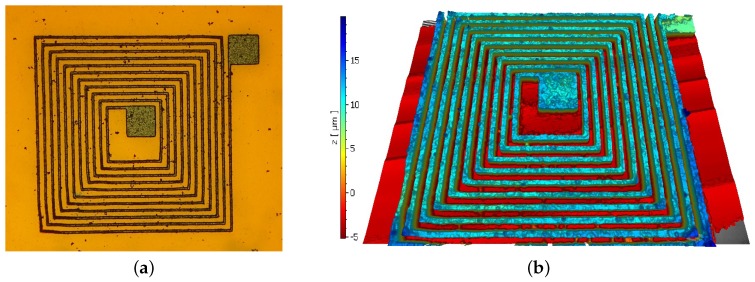
(**a**) Microinductor with 5 μm line width and 20 μm spacing fabricated with condcutive SU-8; (**b**) topography measurement after electroplating.

As an example of miniaturization, we successfully fabricated a microinductor comparable in size with a commercial chip microinductor [29]. It has 5 μm lines with 20 μm spacing in between as shown in Figure 10a. The microcoil was fabricated using the same approach as the NFC antenna, except that the electroplating current was 50 μA for 30 min due to the size of the structure and the thin traces. The electrical resistance of the microinductor was measured in a microprobe station, having a value of 17.9 Ω.

This minimum limit of 5 μm is related to the adhesion between the flexible substrate and the functionalized SU-8 and not to the exposure equipment that has minimum resolution of 0.6 μm, so even smaller traces are feasible on other substrates or when flexibility requirements are lower. The silver nanoparticles embedded in the SU-8 matrix creates a rough surface on the back side of the patterned traces due to light scattering, decreasing its adhesion to the substrate. We noticed during our experiments that longer exposure times led to a better adhesion of conductive tracks to the substrate. A potential application of patterning such small conductive lines would be for the fabrication of fractal RF antennas [24], where a geometry is recursively replicated to increase its effective electrical length in a lower area.

### 3.4. High-Density Interconnections

**Figure 11:**
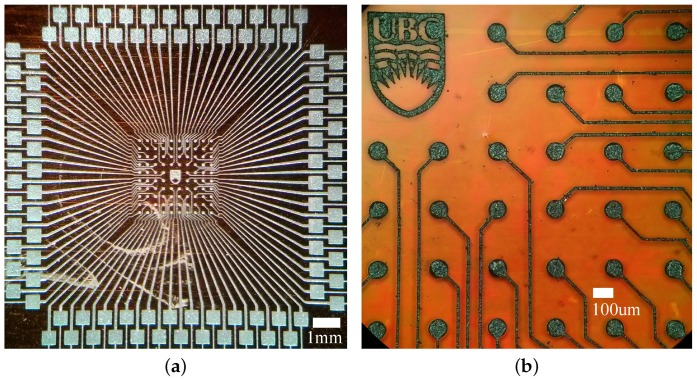
(**a**) BGA fan-out interconnection circuit; (**b**) detailed view of the center of the array.

The proposed fabrication process can be used to pattern high-density interconnection lines comparable to those in the flexible PCB industry [23]. Figure 11 shows an example of a ball grid array (BGA) fan-out circuit, having 96 electrical connections at the center with a pitch of 300 μm; these dimensions are typical of commercial FPGA or microprocessors. It is worth mentioning that the layer thickness obtained with this conductive SU-8 was designed to be thick enough to fabricate MEMS devices with it, observing a strong correlation between viscosity of SU-8 and achievable minimum linewidths.

The average electrical resistivity between the center connections and the external terminals was 35 kΩ; after electroplating the sample for 1.5 h maintaining a current of 1mA, the average resistivity was decreased to 31 Ω. Based on the resistivity values, equivalent interconnections fabricated in gold and platinum would have a resistance 9.4 Ω and 40.7 Ω, respectively, meaning that the resistivity of our electroplated SU-8 structures lies in the same order of magnitude as common metallic materials used for interconnections [30].

### 3.5. LC Resonant Circuit

**Figure 12:**
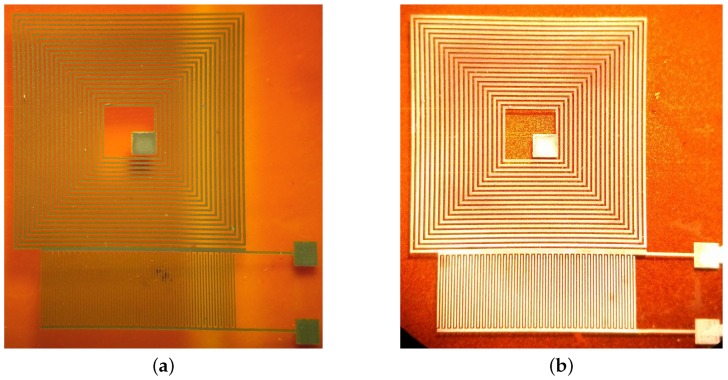
(**a**) Patterned LC circuit before electroplating; (**b**) LC circuit after electroplating, the copper thickness around the conductive SU-8 is 4.5 μm.

**Figure 13:**
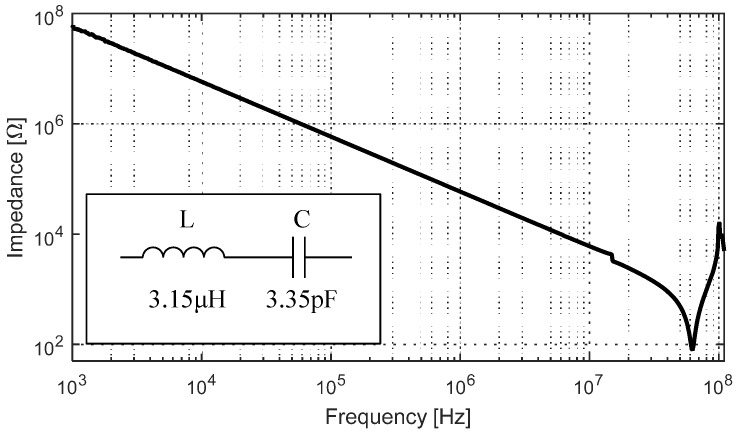
Broad frequency response of the fabricated LC circuit with electroplated copper on top of conductive SU-8 with measured values shown in inset.

We tested the performance of a fully electroplated LC resonator circuit shown in Figure 12. The inductor has 20 turns with 80 μm traces and 80 μm spacing in between. The capacitor has 70 fingers with 50 μm linewidth and 60 μm spacing. After the electroplating process, the spacing between fingers in the capacitor was reduced to 51 μm, increasing the overall capacitance value. The inductance and capacitance values measured independently were 3.15 μH and 3.35 pF, respectively. Figure 13 shows the impedance measurement of the circuit where the resonant and anti-resonant peaks expected of a series LC circuit can be clearly identified.

### 3.6. Bending Effects

Planar devices on flexible substrates are subjected to repeated bending. When a sheet is bent, the outer surface experiences tensile stress and the inner surface compressive stress, while a plane inside the sheet (called the neutral plane) experiences no stress at all. When films are very thin relative to the substrate the simple approximation below describes the relationship between film strain ε and radius of curvature *r*, where *d* is the thickness of the substrate [31],

$$r=\frac{d}{2\varepsilon }.$$

In the case of a film deposited on a substrate, then $d=d_{film}+d_{substrate}$. When the substrate and the film have different Young’s moduli, *Y* and the coated film has a higher value than the substrate, the neutral plane is shifted toward the film reducing the strain by a factor specified in Equation (2) of [32].

Given the process parameters of our samples, we considered a maximum strain of less than 2% for our calculations based on the results of [33]. A correction factor due to the difference of Young’s moduli for 18.5 μm of SU-8 (Y∼ 4 GPa) on a 137 μm Kapton film (Y∼ 2.5 GPa) is 0.944 [32], 4.5 mm being the minimum bending radius to avoid fracture of our tracks.

**Figure 14:**
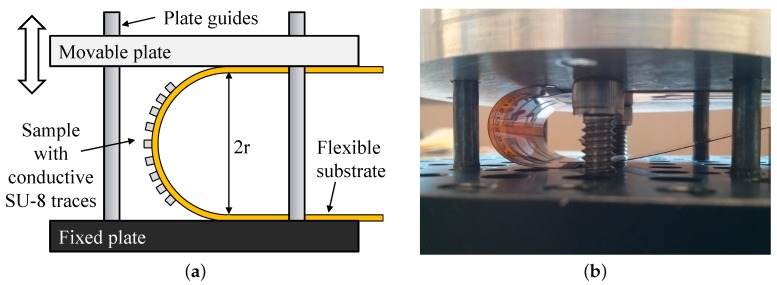
(**a**) Mechanical setup for bending test; (**b**) bending test of LC circuit when bending radius is 15 mm.

To measure the effects of repetitive bending, we created a similar fixture as in [31] shown in Figure 14. The NFC antenna sample presented in Section 3.3 was attached between a fixed and a movable plate guided by metal rods to guarantee a parallel displacement; the radius of curvature is controlled by adjusting mechanical stoppers on the testing bench.

The sample was bent at 15, 10, 5 and 4.5 mm radii for 1, 10, 100 and 1000 times. The average variation in the inductance stayed below 1% from its initial value. For a bending radius below 3 mm, tensile stress caused the conductive tracks to fracture.

**Figure 15:**
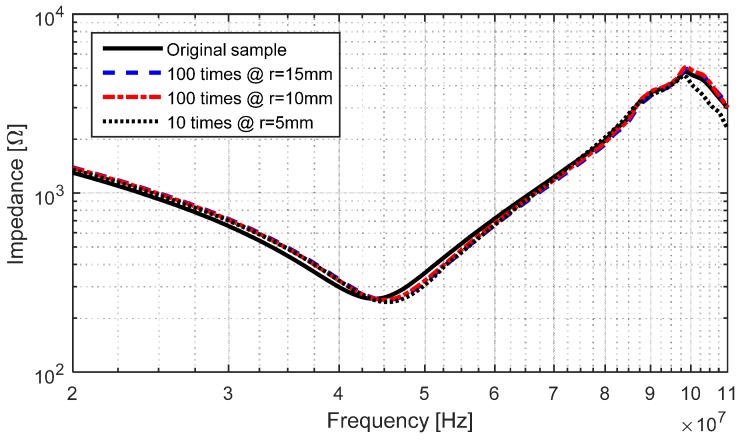
Frequency response of the fabricated LC circuit with electroplated copper on top of conductive SU-8 for different bending radii.

**Figure 16:**
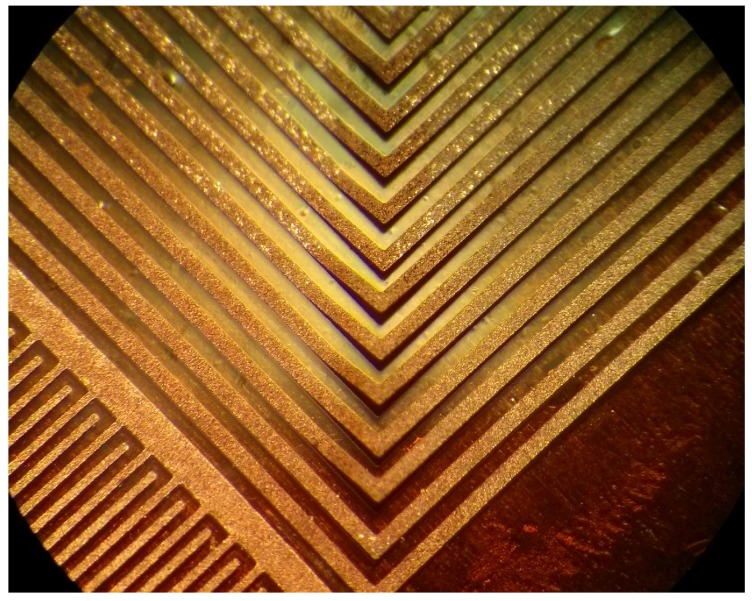
Electroplated copper traces peeling off the substrate after 10 bending cycles when r = 5 mm.

As a complimentary experiment, we tested the effects of compressive stress; we bent the LC circuit sample presented in Section 3.5 for 15, 10 and 5 mm radii. Figure 15 shows the relative insensitivity of the impedance of the circuit after repeated bending; the variation in capacitance and inductance stayed below 2.5%. After bending the sample, 10 cycles at a radius of 5 mm, some of the electrical traces started to peel-off the Kapton substrate as shown in Figure 16, but the sample was still functional; after 100 cycles, several of the traces started to fracture, rendering the sample unusable.

The Kapton film used as substrate shrinks by 0.35% when heated to 200 °C; this low shrinkage percentage on the substrate produced no delamination nor evident damage on the patterned SU-8 layers even after bending; nevertheless, attention is required should substrates with higher shrinkage percentage be used as they might compromise the minimum achievable resolution of patterns; for instance, high-density polyethylene (HDPE) and thermoplastic elastometers (TPE) films exhibit shrinkage rates beyond 2.5%.

It is important to mention that the presented fabrication approach is not limited to flexible substrates; some of the example devices shown were also fabricated on rigid substrates (silicon wafers and glass slides).

## 4. Discussion

We presented the successful fabrication of planar passive devices and circuit interconnects on a flexible piece of Kapton using conductive SU-8 with silver nanoparticles embedded at a concentration of wt 25%. Such components can be the building blocks of more complex sensing schemes such as filters and wireless sensors. To the best of the author’s knowledge, this is the first report of functionalized SU-8 used to fabricate such planar devices and the first on flexible substrates. A demonstration of a miniature inductor design was presented, where conductive line traces as small as 5 μm in width were obtained.

It is worth mentioning that all of the fabricated devices presented in this paper do not have a protective layer on top and are directly exposed to air. The minimum bending radius could potentially be further reduced by adding a protective layer such as parylene or SU-8, thereby enhancing the adhesion of the conductive traces to the substrate. An extra advantage is that these components can easily be manufactured in the inner layers of flexible substrates, occupying less footprint where space is critical in sensing applications.

**Figure 17:**
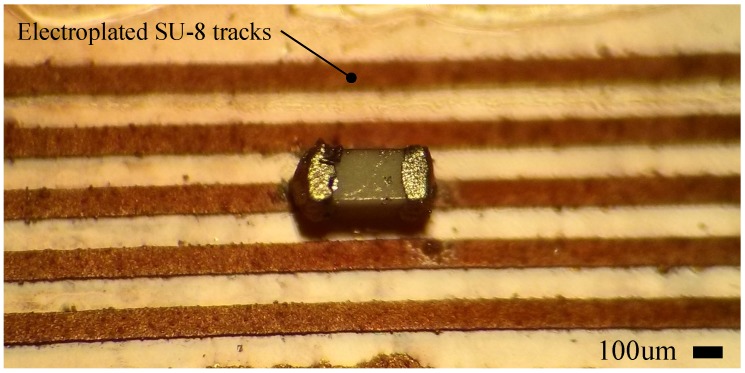
Surface-mounted capacitor soldered between electroplated tracks on functionalized SU-8.

For future work, we plan a hybrid integration of planar devices fabricated with functionalized SU-8, off-the-shelf components and die elements mounted on flexible substrates. As preliminary work, we were able to solder a surface-mounted capacitor between two electroplated tracks on functionalized SU-8 as shown in Figure 17. For future work, we plan to include dielectric materials for multi-layer fabrication of interconnection circuits. 

In summary, this article is meant to highlight the novelty of using conductive SU-8 and electroplating for the fabrication of passive elements and circuit interconnects on flexible substrates. Conductive SU-8 can be used as an alternative to metal deposition when the substrate is sensitive to the environment of metal evaporators and sputtering systems; moreover, functionalized SU-8 can be used as electrical connections for elements that do not require a high electrical conductivity such as capacitive devices. A UV mask aligner was the only required piece of equipment inside a cleanroom to pattern conductive tracks and so it allowed us to continue with the electroplating step in a conventional laboratory using a simple and inexpensive electroplating system, representing a potential reduction in the fabrication costs.
